# The Effect of Cranberry Consumption on C‐Reactive Protein and Interleukin‐6: A Systematic Review and Meta‐Analysis of Randomized Controlled Trials

**DOI:** 10.1002/fsn3.71562

**Published:** 2026-02-16

**Authors:** Mohammad Reza Amini, Mahsa Elahikhah, Sajjad Etesamnia, Motahareh Yadegari, Mohammadreza Moradi Baniasadi, Negin Lohrasbi, Gholamreza Askari

**Affiliations:** ^1^ Nutrition and Food Security Research Center Isfahan University of Medical Sciences Isfahan Iran; ^2^ Department of Nutrition Sciences, School of Paramedical Sciences Ahvaz Jundishapur University of Medical Sciences Ahvaz Iran; ^3^ Department of Clinical Nutrition, School of Nutritional Sciences and Dietetics Tehran University of Medical Sciences (TUMS) Tehran Iran; ^4^ Department of Nutrition, Faculty of Medicine Mashhad University of Medical Sciences Mashhad Iran; ^5^ Department of Community Nutrition, School of Nutritional Sciences and Dietetics Tehran University of Medical Sciences Tehran Iran; ^6^ Student Research Committee, Department of Clinical Nutrition & Dietetics, National Nutrition & Food Technology Research Institute Shahid Beheshti University of Medical Sciences Tehran Iran

**Keywords:** cranberry, C‐reactive protein, interleukin‐6, randomized controlled trials

## Abstract

Previous clinical trials examining the effects of cranberry on inflammatory markers have yielded inconsistent results. This study specifically aimed to assess the influence of cranberry consumption on C‐reactive protein (CRP) and Interleukin‐6 in randomized controlled trials (RCTs). A thorough systematic review was conducted by searching ISI Web of Science, Cochrane Library, PubMed, Scopus, and Google Scholar up to December 21, 2024, with no language restrictions applied, by two independent authors. The results were synthesized using the DerSimonian and Laird random‐effects model. From an initial pool of 1882 articles, 10 were selected for the systematic review and meta‐analysis. The findings indicated that cranberry did not significantly influence CRP (weighted mean differences (WMD): 0.01 mg/L; 95% CI: −0.38 to 0.40, *p* = 0.95; *I* ^2^: 80.8%) or Interleukin‐6 (WMD: −0.26 pg/mL; 95% CI: −1.78 to 1.27, *p* = 0.74; *I* ^2^: 86.1%). However, it was observed that cranberry consumption was associated with a significant rise in CRP levels in obese individuals or in studies focused solely on women. Furthermore, research showed that the active ingredient in cranberry, when administered in powder form, could lead to a considerable increase in interleukin‐6 levels. This review and meta‐analysis suggest that cranberry supplementation did not affect CRP or Interleukin‐6 levels. To further assess and validate these results, more long‐term and well‐designed RCTs are needed.

## Graphical abstract

Cranberry and inflammatory markers.

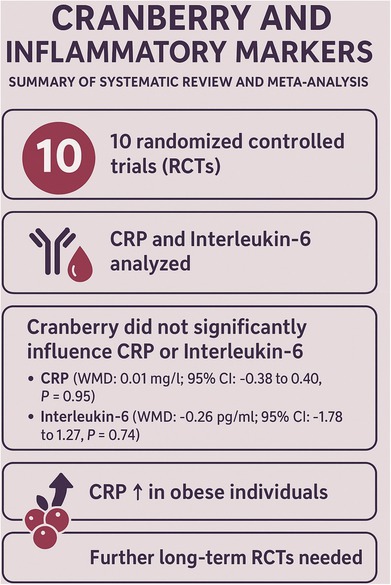

## 1 Introduction

Inflammation is a defense mechanism triggered by harmful factors like pathogens, damaged cells, toxins, irradiation, infection, or tissue injury (Hotamisligil 2017; Medzhitov 2010). Inflammation involves the release of several active signaling compounds, including prostaglandins, leukotrienes, vasoactive amines, complement proteins, and cytokines (Latruffe 2017). Globally, 3 of 5 deaths are caused by chronic inflammatory diseases such as stroke, chronic respiratory conditions, heart disease, cancer, obesity, and diabetes (Barcelos et al. 2019; Tsai et al. 2019). Interleukin 6 (IL‐6), tumor necrosis factor alpha (TNF‐α), and C‐reactive protein (CRP) are key inflammatory markers produced by adipocytes, and their levels are elevated in chronic illnesses (Wellen and Hotamisligil 2005). In recent years, it has been found that dietary patterns and natural products can help alleviate inflammation, which in turn may decrease the burden of diseases (Ahluwalia et al. 2013; Kantor et al. 2013; Paul et al. 2012; Shahavandi et al. 2021).

Cranberries ( *Vaccinium macrocarpon* ) are abundant in flavonoids, such as proanthocyanidins (PAC), anthocyanins, flavanols, and flavonols, as well as phenolic acids like benzoic, hydroxycinnamic, and ellagic acids (McKay and Blumberg 2007). It popularly consumed worldwide in various forms, including fresh and dried fruits, juice, and powdered supplements (Bonifait and Grenier 2010). Cranberry reduced plasma oxidative stress and inflammation (Mathison et al. 2014; Ruel et al. 2008). Also, cranberry decreased plasma Intercellular Adhesion Molecule 1 (ICAM‐1) and vascular cell adhesion molecule 1 (VCAM‐1) concentrations (Ruel et al. 2008). Studies have demonstrated that cranberries and their components exhibit antibacterial (Leitão et al. 2005), antiviral (Weiss et al. 2005), anticarcinogenic (Sun and Hai Liu 2006), and antiangiogenic (Roy et al. 2002) properties.

Several studies investigated the effect of cranberry in different forms on inflammation. Most studies indicated that cranberry did not improve inflammatory biomarkers such as IL‐6 or CRP (Chew et al. 2019; Dohadwala et al. 2011; Lee et al. 2008; Richter et al. 2021). However, a few studies showed promising results which decreased inflammation (Fatel et al. 2021; Novotny et al. 2015). Additionally, randomized controlled trials (RCTs) were conducted using various forms of cranberry, including supplements (Eftekhari et al. 2016), juice (Dohadwala et al. 2011; Richter et al. 2021), and extract (Lee et al. 2008). These controversies provide a reason to conduct a comprehensive systematic review and meta‐analysis to evaluate the effects of cranberry consumption on IL‐6 and CRP in adults.

## 2 Materials and Methods

The present systematic review and meta‐analysis was designed and conducted in accordance with the Preferred Reporting Items for Systematic Reviews and Meta‐Analyses (PRISMA) guidelines (Liberati et al. 2009).

### 2.1 Search Strategy

A thorough examination of the scientific literature was conducted to evaluate the effects of cranberry consumption on CRP and IL‐6. We systematically searched several databases, including ISI Web of Science, Cochrane Library, Embase, UpToDate, PubMed, and Scopus, to identify all relevant studies. The search was conducted up to December 21, 2024, utilizing keywords and MeSH terms such as *Vaccinium microcarpum* , *V. macrocarpon* , *Vaccinium erythrocarpum* , RCTs, and placebo. Furthermore, we manually reviewed relevant article references as well as the top 500 articles from Google Scholar. There were no restrictions on the time frame or language of the studies, and all identified studies were included in the screening process.

### 2.2 Study Selection

The inclusion criteria for this analysis consisted of comparative studies investigating the effects of cranberry consumption on CRP and/or IL‐6. Reviewers should primarily prioritize RCTs that involve participants aged 18 years and older. Additionally, the articles must detail the frequency, dosage, and duration of the intervention. It is essential to note that only studies meeting these specific criteria were included in the analysis; any studies that did not conform to these standards were excluded. The exclusion criteria encompassed animal studies, previously conducted systematic reviews or meta‐analyses, and non‐randomized studies. Additionally, studies involving pregnant or lactating women, interventions with a duration of < 2 weeks, complex interventions, and studies lacking critical data were excluded from consideration. The PICOS criteria were established as follows: the inclusion of adult participants; an intervention involving cranberry consumption; a comparative group that received no intervention; outcome measures encompassing CRP and IL‐6; and an RCT design.

### 2.3 Data Extraction and Assessment of Risk of Bias

Data required for synthesis and analysis were systematically extracted from each article by two independent investigators (NL, MY). Any discrepancies encountered during the research phase were addressed through open dialogue and negotiation between the investigators until a consensus was achieved. In the meta‐analysis, we collected and reported mean and standard deviations (SDs) values for CRP and IL‐6 levels both before and after the intervention in the intervention and control groups, thereby assessing the impact of cranberry consumption on these biomarker levels. Furthermore, the mean and SDs of changes in CRP and IL‐6 levels for each group were computed. Additionally, we documented relevant information, including author names, study sites, year of publication, sample size, mean age, health status, study design, intervention dose and duration, comparison groups, and type of intervention. CRP and IL‐6 levels were converted to international units, from which we calculated the mean difference and SDs of changes.

Reviewers applied the Cochrane Risk of Bias Tool to standardize the included articles and identify potential biases, ensuring the integrity of the review process (Higgins et al. 2011). Each component of the tool was evaluated based on the information provided in the articles, with scores assigned to indicate the level of bias risk as high, low, or unclear. The tool assesses various factors in the included articles, such as random sequence generation, allocation concealment, selective reporting, and blinding of both participants and personnel, as well as the blinding of outcome assessment, incomplete outcome data, and other potential sources of bias. When all domains are evaluated, if they consistently indicate a low risk of bias, the overall assessment of the study is deemed to be at low risk of bias. If at least one domain is rated as unclear but shows no indication of bias, the overall score is classified as unclear. Conversely, if any domain indicates a high risk of bias, the study is considered to be at high risk of bias overall (Sterne et al. 2019).

### 2.4 Statistical Analysis

To evaluate the overall impact of cranberry on CRP and IL‐6 levels, we calculated the mean differences and SDs for all relevant outcomes. In instances where the mean and SD were not provided, we computed the values to express them as mean (SD). The results of the analysis were displayed as bias‐corrected weighted mean differences (WMD) and 95% confidence intervals (CI). The mean changes for each variable were calculated by subtracting post‐intervention values from pre‐intervention values, separately for both the intervention and control groups, and this formula was used to calculate the SD changes for each variable in both the intervention and control groups while considering the correlation coefficient (r) of 0.8: “[(SD1)2 + (SD2)2] − [ 2 × *r* × SD1 × SD2]” (Cumpston et al. 2019). A random‐effects model employing the Der Simonian and Laird method was implemented to estimate the overall effect size while accounting for the potential heterogeneity present among the studies incorporated in the analysis (DerSimonian and Laird 1986). To evaluate heterogeneity across studies, both the *I* ^2^ and Cochran's *Q*‐test were employed. A study was deemed to exhibit significant heterogeneity if the *I* ^2^ value was more than 50% or if the *p*‐value derived from Cochran's *Q*‐test was < 0.05.

Subgroup analyses were performed to identify sources of heterogeneity and provide detailed descriptions of outcomes by age, health status, intervention dose, and duration. Sensitivity analyses were performed to assess the impact of individual studies on the overall study results. Symmetry in the funnel plot indicates the absence of publication bias. For statistical analysis, Begg's rank correlation test and Egger's regression asymmetry test were used. All analyses were conducted using STATA, version 14 (Stata Corp, College Station, Texas, USA), and *p*‐values below 0.05 were considered statistically significant.

## 3 Results

### 3.1 Literature Search and Study Characteristics

**FIGURE 1:**
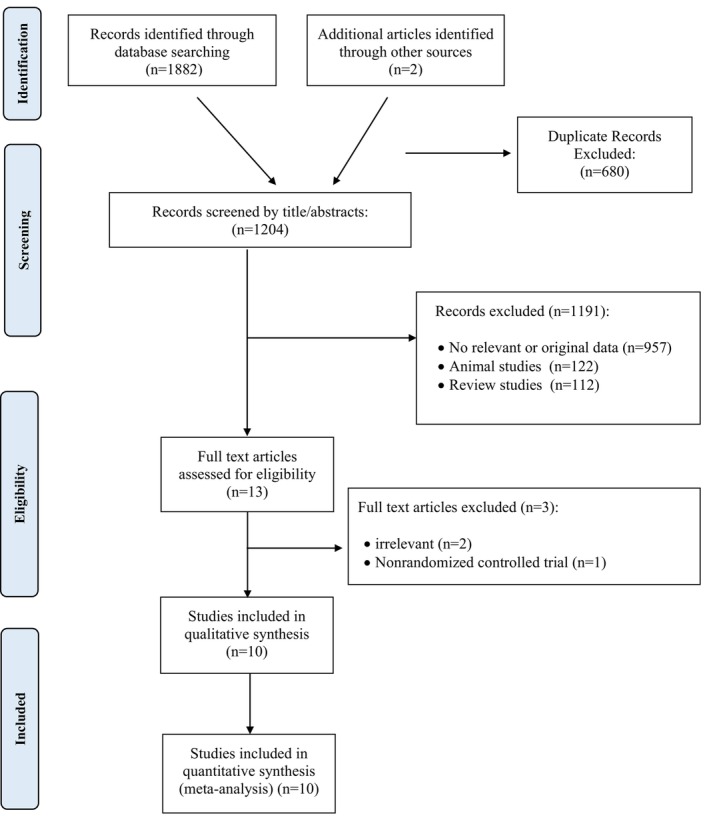
Flow chart of the number of studies identified and selected into the meta‐analysis.

Following a comprehensive initial systematic search, we identified 1882 studies across various databases. In addition, we obtained two studies from a manual search. We excluded 680 duplicate studies, which were identified as overlapping articles across multiple databases, retaining only a single copy of each. After the removal of duplicates, 1204 studies advanced to the screening stage. A comprehensive review of the titles and abstracts resulted in the exclusion of 957 studies, as they were either unrelated to the research topic or failed to present new and original data for analysis. Furthermore, since this meta‐analysis was exclusively focused on human studies, 122 animal studies and 112 review studies were also excluded. In the end, 13 articles were retained for full‐text review. After a comprehensive evaluation of these articles, two were excluded due to their continued irrelevance to the primary research topic. Since meta‐analysis commonly necessitates RCTs to ensure result accuracy, one non‐randomized study that did not fulfill the inclusion criteria was also excluded (Simão et al. 2013). Upon the completion of all screening and eligibility assessments, a total of 10 studies were deemed appropriate for inclusion in the qualitative and quantitative analysis (Figure 1).

The Cochrane Risk of Bias tool evaluated potential biases across various domains. All studies indicated a low risk of bias in terms of random sequence generation. However, the allocation concealment was unclear in seven of the studies (Basu et al. 2011; de Souza Gouveia Moreira et al. 2024; Dohadwala et al. 2011; Fatel et al. 2021; Flammer et al. 2013; Lee et al. 2008; Novotny et al. 2015). One study was deemed to have a high risk of performance bias due to the absence of blinding for both participants and personnel (Fatel et al. 2021). Furthermore, the blinding status of outcome assessors remained unclear in eight studies, raising concerns about detection bias (Chew et al. 2019; de Souza Gouveia Moreira et al. 2024; Dohadwala et al. 2011; Eftekhari et al. 2016; Flammer et al. 2013; Lee et al. 2008; Novotny et al. 2015; Richter et al. 2021). Fortunately, all studies provided comprehensive reports of their results, which helps mitigate the risk of incomplete outcome data (Table 1).

**TABLE 1:** Risk of bias for randomized controlled trials, assessed according to the Revised Cochrane risk‐of‐bias tool for randomized trials.

Publications	Random sequence generation	Allocation concealment	Selective reporting	Blinding (participants and personnel)	Blinding (outcome assessment)	Incomplete outcome data	Other source of bias
1. Basu et al. (2011)	L	U	L	L	L	L	L
2. Chew et al. (2019)	L	L	L	L	U	L	L
3. Dohadwala et al. (2011)	L	U	L	L	U	L	L
4. Eftekhari et al. (2016)	L	L	L	L	U	L	L
5. Fatel et al. (2021)	L	U	L	H	H	L	L
6. Flammer et al. (2013)	L	U	L	L	U	L	L
7. de Souza Gouveia Moreira et al. (2024)	L	U	L	L	U	L	H
8. Lee et al. (2008)	L	U	L	L	U	L	L
9. Novotny et al. (2015)	L	U	L	L	U	L	L
10. Richter et al. (2021)	L	L	L	L	U	L	L

Abbreviations: H, high risk of bias; L, low risk of bias; U, unknown.

All ten studies included in the analysis were designed as RCTs. Three of these employed a crossover design (Chew et al. 2019; Dohadwala et al. 2011; Richter et al. 2021), while seven utilized a parallel design (Basu et al. 2011; de Souza Gouveia Moreira et al. 2024; Eftekhari et al. 2016; Fatel et al. 2021; Flammer et al. 2013; Lee et al. 2008; Novotny et al. 2015). Six studies were conducted in the United States (Basu et al. 2011; Chew et al. 2019; Dohadwala et al. 2011; Flammer et al. 2013; Novotny et al. 2015; Richter et al. 2021), two in Brazil (de Souza Gouveia Moreira et al. 2024; Fatel et al. 2021), one in Iran (Eftekhari et al. 2016), and one in Taiwan (Lee et al. 2008). Two of the studies focused exclusively on women (Basu et al. 2011; Eftekhari et al. 2016), while the remaining eight included participants of both genders (Chew et al. 2019; de Souza Gouveia Moreira et al. 2024; Dohadwala et al. 2011; Fatel et al. 2021; Flammer et al. 2013; Lee et al. 2008; Novotny et al. 2015; Richter et al. 2021). Out of the total studies, seven reported both CRP and IL‐6 indices (Basu et al. 2011; Chew et al. 2019; de Souza Gouveia Moreira et al. 2024; Eftekhari et al. 2016; Fatel et al. 2021; Flammer et al. 2013), whereas four studies examined only CRP (Dohadwala et al. 2011; Lee et al. 2008; Novotny et al. 2015; Richter et al. 2021). Among these, two studies focused on individuals with metabolic syndrome (Basu et al. 2011; Eftekhari et al. 2016), three on participants with cardiovascular disorder (Dohadwala et al. 2011; Flammer et al. 2013; Richter et al. 2021), two on healthy individuals (Chew et al. 2019; Novotny et al. 2015), one on people with type 2 diabetes (Lee et al. 2008), one on patients with chronic kidney disease (de Souza Gouveia Moreira et al. 2024), and one on individuals with rheumatoid arthritis (Fatel et al. 2021). The studies included a total of 462 participants, with an average age of 52.2 years. Of these, seven studies investigated the effects of cranberry juice (Basu et al. 2011; Chew et al. 2019; Dohadwala et al. 2011; Fatel et al. 2021; Flammer et al. 2013; Novotny et al. 2015; Richter et al. 2021), while three utilized cranberry powder (de Souza Gouveia Moreira et al. 2024; Eftekhari et al. 2016; Lee et al. 2008). The intervention periods ranged from 4 to 16 weeks. The articles featured in this review were published between 2008 and 2024 (Table 2).

**TABLE 2:** Demographic characteristics of the included studies.

First author (year)	Location	Study design	Health status	Sex	Sample size	Duration (week)	Mean age (year)	Baseline BMI (kg/m^2^)	Intervention group	Comparator group	Outcome
1. Basu et al. (2011)	USA	RCT, parallel	Metabolic syndrome	Female	31	8	52	40	480 mL low‐calorie cranberry juice	Placebo	CRP/IL‐6
2. Chew et al. (2019)	USA	RCT, crossover	Healthy	Both	78	8	50	31	480 mL low‐calorie cranberry extract beverage (CEB)	Placebo	CRP/IL‐6
3. Dohadwala et al. (2011)	USA	RCT, crossover	Coronary artery disease	Both	44	4	62	29.5	480 mL cranberry juice, double‐strength (54% juice)	Placebo	CRP
4. Eftekhari et al. (2016)	Iran	RCT, parallel	Metabolic syndrome	Female	48	8	42	29.3	400 mg cranberry supplement	Placebo	CRP/IL‐6
5. Fatel et al. (2021)	Brazil	RCT, parallel	Rheumatoid arthritis	Both	41	12	58	26.7	500 mL reduced‐calorie cranberry juice and 3 g of fish oil n‐3 fatty acids	3 g of fish oil n‐3 fatty acids	CRP/IL‐6
6. Flammer et al. (2013)	USA	RCT, parallel	Peripheral endothelial dysfunction and cardiovascular risk factors	Both	69	16	49.5	27.4	460 mL cranberry juice cocktail	Placebo	CRP/IL‐6
7. de Souza Gouveia Moreira et al. (2024)	Brazil	RCT, parallel	Chronic kidney disease (stages 3–4)	Both	25	8	57.7	29.7	1 g cranberry extract	Placebo	CRP/IL‐6
8. Lee et al. (2008)	Taiwan	RCT, parallel	Type 2 diabetes	Both	30	12	65.5	26	500 mg cranberry extract	Placebo	CRP
9. Novotny et al. (2015)	USA	RCT, parallel	Healthy	Both	56	8	50	28	480 mL low‐calorie cranberry juice	Placebo	CRP
10. Richter et al. (2021)	USA	RCT, crossover	Elevated blood pressure	Both	40	8	47	28.7	500 mL cranberry juice	Placebo	CRP

Abbreviations: BMI, body mass index; CRP, C‐reactive protein; IL‐6, interleukin 6; RCT, randomized controlled trial.

### 3.2 Findings From Meta‐Analysis

**FIGURE 2:**
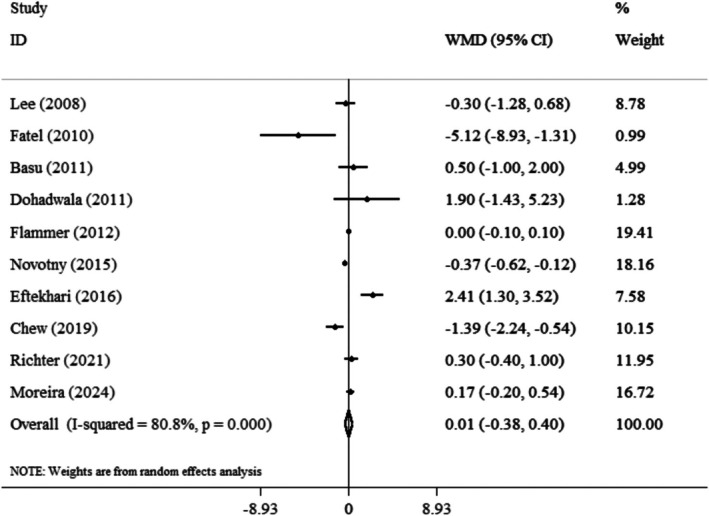
Forest plot detailing weighted mean difference and 95% confidence intervals (CIs) for the effect of cranberry consumption on CRP.

**FIGURE 3:**
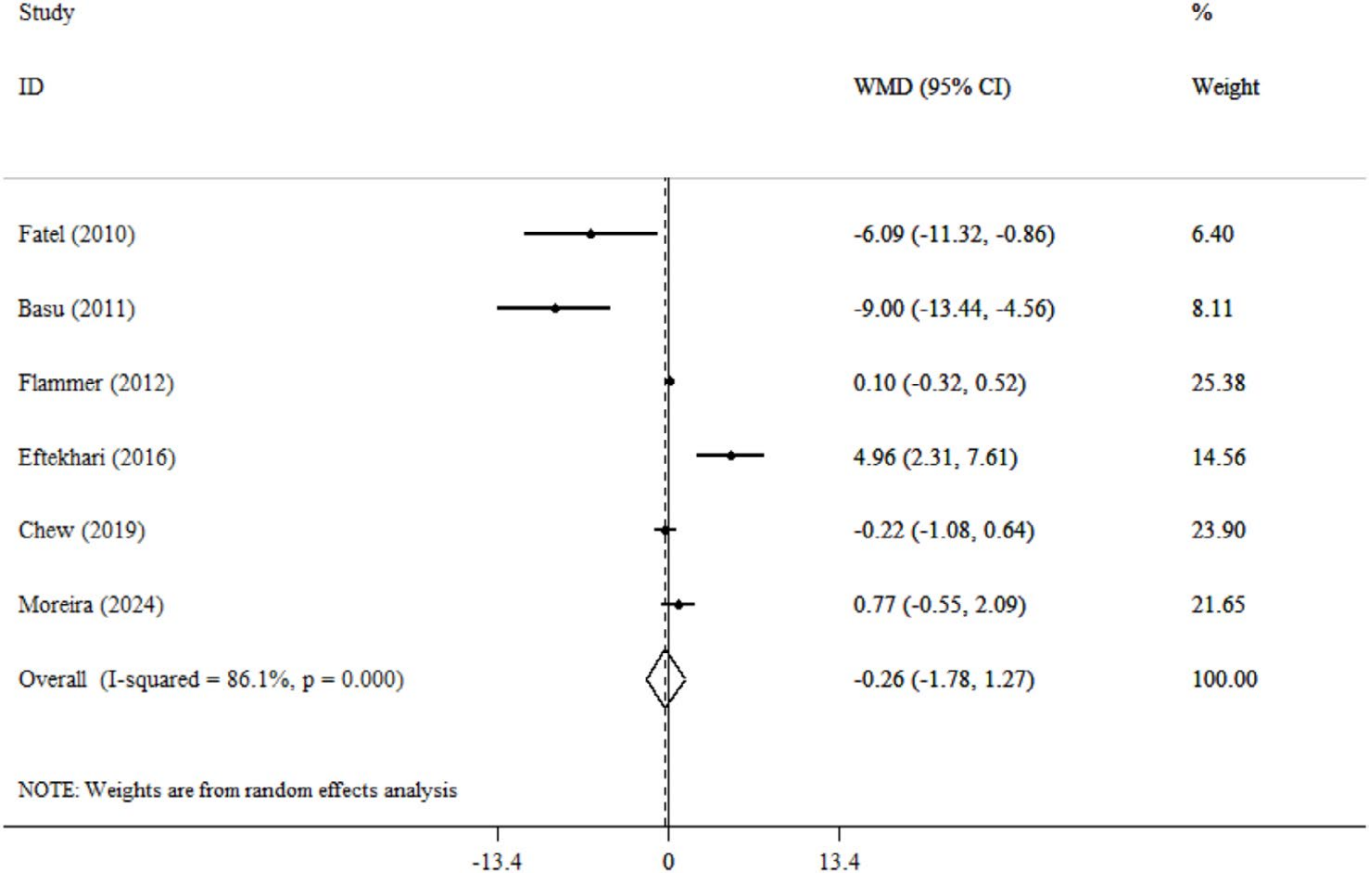
Forest plot detailing weighted mean difference and 95% confidence intervals (CIs) for the effect of cranberry consumption on IL‐6.

**FIGURE 4:**
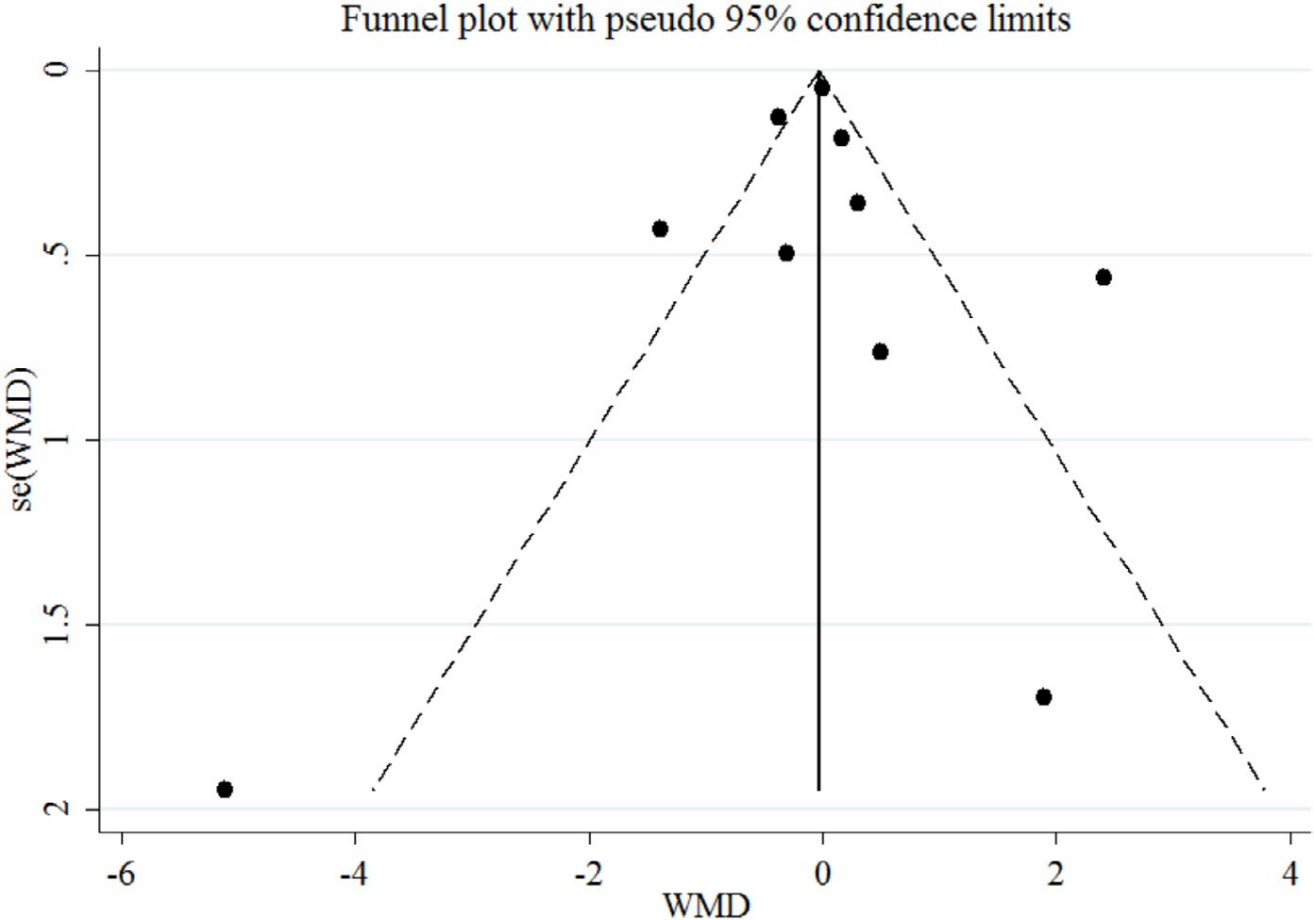
Funnel plot displaying publication bias in the studies reporting the impact of cranberry consumption on CRP.

The final analysis, which reviewed 10 studies to assess the impact of cranberry consumption on CRP, concluded that there was no significant relationship between cranberry intake and CRP levels (WMD: 0.01 mg/L; 95% CI: −0.38, 0.40, *p* = 0.95; *I* ^2^: 80.8%) (Figure 2). Furthermore, an analysis of six studies indicated that cranberry consumption did not have a significant effect on IL‐6 levels (WMD: −0.26 pg/mL; 95% CI: −1.78, 1.27, *p* = 0.74; *I* ^2^: 86.1%) (Figure 3). Although the pooled estimates for both CRP and IL‐6 did not reach statistical significance, the overall direction of effect across most studies showed a modest tendency toward reduced inflammatory marker levels, with six of the 10 included trials favoring the cranberry group. These observations may reflect biological variability rather than a true lack of effect. The considerable heterogeneity observed (*I* ^2^ > 80%) highlights differences in study populations, dosage forms, and intervention durations. Studies using cranberry juice tended to show smaller changes in inflammatory markers compared with those using cranberry powder, possibly due to differences in bioavailability and phenolic content. Differences in participants' health status also contributed to this variability, with trials including obese or metabolically unhealthy participants showing more pronounced responses. Subgroup analysis was employed to identify sources of heterogeneity in the articles and to provide a clearer description of the results, focusing on age, gender, duration of intervention, and type of intervention (powder and juice) (Table 3). A subgroup analysis based on body mass index (BMI) found that individuals classified as obese, with a BMI exceeding 30, exhibited significantly reduced levels of CRP (WMD: −0.93 mg/L; 95% CI: −1.67, −0.19, *p* = 0.01) (Figure 4). Additionally, the analysis by gender revealed that studies focused solely on women indicated a significant increase in CRP levels associated with cranberry consumption (WMD: 1.74 mg/L; 95% CI: 0.85, 2.63, *p* = 0.001). Moreover, research demonstrated that the active ingredient, when administered in powder form, can lead to a substantial rise in interleukin‐6 levels (WMD: 1.61 pg/mL; 95% CI: 0.43, 2.79, *p* = 0.008). These subgroup results suggest that individual characteristics may strongly influence the anti‐inflammatory effects of cranberry. The reduction in CRP among obese individuals may reflect their higher baseline inflammation, which makes them more responsive to polyphenol‐rich interventions. Conversely, the elevation in CRP among women might be explained by hormonal and metabolic factors affecting inflammatory pathways. The increase in IL‐6 levels observed with cranberry powder could be linked to higher doses of active compounds or to differences in preparation methods, such as drying and concentration processes, which alter the phenolic profile. In the context of sensitivity analysis, each study was systematically removed from the dataset to assess its impact on the overall results. The findings showed that excluding any individual study did not significantly change the overall conclusions for CRP and IL‐6. To assess publication bias, the funnel plot revealed a symmetrical distribution of study effects. Furthermore, two statistical tests were conducted to evaluate publication bias: Egger's regression asymmetry test and Begg's rank correlation test. The outcomes were 0.98 and 0.78 for CRP, and 0.64 and 0.57 for IL‐6, respectively. These results indicate that there was no statistical evidence of publication bias in this study.

**TABLE 3:** Subgroup analysis of included randomized controlled trials in meta‐analysis of the effect of cranberry consumption on inflammation.

Group	No. of trials	WMD (95% CI)	*p*	*I* ^2^ (%)	P‐heterogeneity	*P* for between subgroup heterogeneity
*CRP*						
Type of intervention						0.03
Powder	3	0.31 (−0.01, 0.64)	0.06	87.4	< 0.001	
Juice	7	−0.06 (−0.15, 0.03)	0.20	77.2	< 0.001	
Duration (week)						0.27
≤ 8	7	−0.13 (−0.32, 0.06)	0.19	84.3	< 0.001	
> 8	3	−0.01 (−0.10, 0.09)	0.89	77.5	0.02	
Age						0.38
≤ 50	5	−0.04 (−0.13, 0.05)	0.35	89.1	< 0.001	
> 50	5	0.11 (−0.22, 0.44)	0.52	57.3	0.05	
Mean BMI						0.01
25–29.9	8	−0.02 (−0.11, 0.07)	0.66	80.8	< 0.001	
≥ 30	2	−0.93 (−1.67, −0.19)	0.01	78.3	0.03	
Sex						< 0.001
Both	8	−0.05 (−0.14, 0.04)	0.27	74.5	< 0.001	
Female	2	1.74 (0.85, 2.63)	< 0.001	72.5	0.04	
*IL‐6*						
Type of intervention						0.009
Powder	2	1.61 (0.43, 2.79)	0.008	87.0	0.006	
Juice	4	−0.05 (−0.43, 0.32)	0.77	86.0	< 0.001	
Duration (week)						0.75
≤ 8	4	0.19 (−0.50, 0.88)	0.59	90.2	< 0.001	
> 8	2	0.06 (−0.35, 0.48)	0.77	81.3	0.02	
Age						0.44
≤ 50	3	0.14 (−0.23, 0.51)	0.47	85.1	0.001	
> 50	3	−0.37 (−1.60, 0.87)	0.56	90.9	< 0.001	
Mean BMI						0.10
25–29.9	4	0.23 (−0.16, 0.62)	0.24	84.1	< 0.001	
≥ 30	2	−0.54 (−1.38, 0.31)	0.21	93.1	< 0.001	
Sex						0.29
Both	4	0.06 (−0.29, 0.42)	0.72	56.2	0.07	
Female	2	1.30 (−0.97, 3.57)	0.26	96.4	< 0.001	

Abbreviations: BMI, body mass index; CRP, C‐reactive protein; IL‐6, interleukin 6; WMD, weight mean difference.

## 4 Discussion

Based on the findings of the current meta‐analysis, cranberry consumption does not lead to a significant reduction in CRP or IL‐6 levels. Subgroup analyses revealed that cranberry consumption significantly reduced CRP levels in individuals with BMI ≥ 30. However, an inverse effect was observed in female participants, where cranberry consumption led to a significant increase in CRP levels. Additionally, the consumption of cranberry in powdered form was associated with a significant elevation in IL‐6 levels.

Cranberry ( *V. macrocarpon* ), are highly valued for their rich antioxidant content, low carbohydrates, and high levels of vitamins, minerals, and phenolic compounds (Simão et al. 2013; Vinson et al. 2008). They contain a unique mix of flavonoids, catechins, and phenolic acids, which contribute to antioxidant effects, enzyme modulation, and gene regulation (Mathison et al. 2014; McKay and Blumberg 2007; McKay et al. 2015; Neto 2007). Studies using animal models demonstrate that cranberry extracts can lower levels of C‐reactive protein and inflammatory interleukins, and enhance the production of nitric oxide (Kim et al. 2013, 2011; Zhu et al. 2011). Recent studies indicate that chronic inflammation plays a crucial role in the development of various diseases such as cancer, cardiovascular conditions, diabetes, insulin resistance, and rheumatoid arthritis (Libby 2006; Moss and Blaser 2005; Shoelson et al. 2006). Nuclear factor kappa B (NF‐κB) is a key transcription factor that activates genes responsible for producing cytokines, chemokines, and other molecules involved in the inflammatory response (Pahl 1999). Our study did not show a significant reduction in CRP and IL‐6 levels with cranberry consumption, but cranberries are thought to have anti‐inflammatory effects due to their high polyphenol content. In vitro studies suggest that cranberry compounds can inhibit macrophage and T cell activation, even under strong inflammatory conditions, indicating a need for further research to explore their benefits (Bodet et al. 2006). Polyphenols, including resveratrol found in cranberries, have been shown in cell culture studies to suppress inflammatory genes by affecting transcription factors such as NF‐κB and JAK/STAT (McKay and Blumberg 2007). Denis et al. (2015) found that cranberry fractions reduced NF‐κB activation and lowered levels of pro‐inflammatory mediators such as TNF‐α and IL‐6 in Caco‐2/15 intestinal cells, even under LPS‐induced inflammation (Denis et al. 2015). A decrease in COX2 expression was noted, which is associated with reduced PGE2 levels. Cranberry extract supplementation in male rats was associated with a reduced expression rate of NF‐κB (Anhê et al. 2015). In contrast to the existing evidence, some studies attempting to demonstrate the anti‐inflammatory effects of cranberries failed to yield positive outcomes regarding inflammatory markers. Simão et al. (2013) found no alteration in NF‐κB expression following a 60‐day intervention with cranberry juice (Simão et al. 2013). Additionally, individuals with metabolic syndrome experienced no changes in CRP and IL‐6 levels after consuming 480 mL of cranberry juice daily for 8 weeks (Basu et al. 2011). Cranberry polyphenols have been shown to reduce inflammation by suppressing NF‐κB expression, which decreases the production of inflammatory cytokines. The impact of polyphenols on cell signaling pathways is evident, particularly in their potential to modulate inflammatory pathways (Choi et al. 2004; Collett and Campbell 2004; Kunnumakkara et al. 2007). Polyphenols influence inflammation through both indirect and direct mechanisms. They alter the cellular redox state and directly inhibit receptors and transcription factors like TLR‐4, NF‐κB, AP‐1, and JNK, reducing proinflammatory gene expression (e.g., interleukins). Additionally, they may act as natural ligands for PPAR‐γ, further regulating inflammatory pathways (Choi et al. 2004; Collett and Campbell 2004; Joseph et al. 2016; Kunnumakkara et al. 2007; Santangelo et al. 2007). We have presented several examples of conflicting results regarding the effect of cranberry consumption on reducing CRP and IL‐6 levels. For instance, Basu et al. (2011) found that low‐calorie cranberry juice increased antioxidant capacity and reduced lipid oxidation markers in women with metabolic syndrome, but did not affect CRP or IL‐6 levels (Basu et al. 2011). In contrast, Fatel et al. (2021) study involving 62 people with rheumatoid arthritis showed that combining cranberry juice with fish oil significantly reduced CRP and IL‐6 levels, outperforming fish oil alone (Fatel et al. 2021). However, de Souza Gouveia Moreira et al. (2024) found no significant impact of cranberry extract supplementation on CRP or IL‐6 levels in patients with chronic kidney disease (de Souza Gouveia Moreira et al. 2024). These studies highlight the variability in cranberry's effects on inflammation markers, suggesting that outcomes may depend on specific conditions, combinations with other supplements, or study designs.

The current meta‐analysis indicates that cranberry consumption does not significantly reduce CRP or IL‐6 levels, possibly due to variability in study designs, dosages, and participant characteristics. Other confounding factors, such as participants' dietary and lifestyle habits, may also have influenced inflammation levels, making it difficult to isolate the effects of cranberry. While some studies report anti‐inflammatory benefits of cranberries, others show no significant impact, highlighting the need for further research to clarify their role in modulating inflammatory markers like CRP and IL‐6 (de Souza Gouveia Moreira et al. 2024; Wang et al. 2022). Subgroup analyses reveal that cranberry consumption significantly reduces CRP levels in individuals with BMI ≥ 30, likely due to the heightened inflammatory state associated with obesity. Obese individuals often exhibit elevated baseline CRP levels, which makes them more responsive to anti‐inflammatory interventions. Cranberry's rich polyphenolic content, known for its anti‐inflammatory properties, appears to modulate inflammation by reducing NF‐ĸB activation and lowering pro‐inflammatory cytokines such as TNF‐α and IL‐6. Studies have also shown that cranberry consumption improves oxidative stress biomarkers and lipid profiles, which are often disrupted in obese individuals with chronic low‐grade inflammation. These mechanisms collectively explain why cranberry consumption has a more pronounced impact on reducing CRP levels in this population subset (Chew et al. 2019; Duffey and Sutherland 2015; Hsia et al. 2020). Elevated CRP levels in female cranberry consumers may stem from gender‐specific biological and physiological factors. Hormonal variations between sexes could alter inflammatory responses to dietary interventions, as studies suggest women exhibit distinct inflammatory profiles compared to men—particularly when consuming polyphenol‐rich foods like cranberries (Hsia et al. 2020; Novotny et al. 2015). The observed elevation in IL‐6 levels associated with cranberry powder consumption in some studies may arise from context‐dependent factors, such as dosage, experimental conditions, or population‐specific responses. For instance, while most studies indicate that cranberry powder reduces IL‐6 in models of inflammation (e.g., LPS‐induced inflammation in rats; Kim et al. 2011), variations in cranberry formulation or metabolic interactions could lead to divergent outcomes.

This systematic review and meta‐analysis possess both notable limitations and strengths that warrant consideration. To our knowledge, this is the first meta‐analysis evaluating the effects of cranberry consumption on CRP and IL‐6. Second, to ensure the most comprehensive systematic review and meta‐analysis, we incorporated all eligible articles without restrictions on language or publication date, thereby enhancing the generalizability of the findings. Additionally, our study demonstrated high internal validity and was unaffected by publication bias, given that all included studies were conducted as randomized, double‐blind, placebo‐controlled trials. Furthermore, we performed sensitivity analyses, which enhanced the robustness and power of our analysis. However, this article is subject to several limitations that need to be acknowledged. First, significant heterogeneity existed among the studies that were included. In the subgroup analysis, factors such as type of intervention, duration, age, mean BMI, and sex could explain the variation between studies. Second, as no additional data are available for subgroup analysis, caution is warranted when applying the current findings to different demographic groups. Genetic factors may influence the efficacy of cranberry consumption, but we could not assess this possibility due to variations in the study populations. The findings were constrained by the limited range of participants' health conditions and the small sample sizes in some of the studies. In light of these limitations, further primary clinical trials with larger sample sizes and more sophisticated designs are needed to validate these findings.

In conclusion, cranberry consumption does not significantly lower IL‐6 or CRP levels. Cranberry eating significantly lowered CRP levels in people with a BMI of 30 or higher, according to subgroup analyses. In contrast, cranberry eating significantly raised CRP levels in female subjects, exhibiting an opposing effect. Furthermore, a substantial increase in IL‐6 levels was linked to the ingestion of powdered cranberries. Future studies should focus on large‐scale, long‐term RCTs using standardized cranberry formulations to minimize variability across interventions.

## Author Contributions

**Mahsa Elahikhah, Sajjad Etesamnia, and Mohammadreza Moradi Baniasadi:** writing – original draft. **Motahareh Yadegari:** methodology, writing – original draft. **Negin Lohrasbi:** data curation, formal analysis, methodology. **Gholamreza Askari:** writing – review editing. **Mohammad Reza Amini:** supervision, writing – review and editing.

## Funding

This work was supported by the Nutrition and Food Security Research Center, Isfahan University of Medical Sciences, Isfahan, Iran (no. 2404250).

## Conflicts of Interest

The authors declare no conflicts of interest.

## Data Availability

The data used to support the findings of this study are available from the corresponding author upon request.

## References

[fsn371562-bib-0001] Ahluwalia, N. , V. A. Andreeva , E. Kesse‐Guyot , and S. Hercberg . 2013. “Dietary Patterns, Inflammation and the Metabolic Syndrome.” Diabetes & Metabolism 39, no. 2: 99–110. 10.1016/j.diabet.2012.08.007. 23062863

[fsn371562-bib-0002] Anhê, F. F. , D. Roy , G. Pilon , et al. 2015. “A Polyphenol‐Rich Cranberry Extract Protects From Diet‐Induced Obesity, Insulin Resistance and Intestinal Inflammation in Association With Increased Akkermansia spp. Population in the Gut Microbiota of Mice.” Gut 64, no. 6: 872–883. 10.1136/gutjnl-2014-307142. 25080446

[fsn371562-bib-0003] Barcelos, I. P. , R. M. Troxell , and J. S. Graves . 2019. “Mitochondrial Dysfunction and Multiple Sclerosis.” Biology‐Basel 8, no. 2: 37. 10.3390/biology8020037. 31083577 PMC6627385

[fsn371562-bib-0004] Basu, A. , N. M. Betts , J. Ortiz , B. Simmons , M. Wu , and T. J. Lyons . 2011. “Low‐Energy Cranberry Juice Decreases Lipid Oxidation and Increases Plasma Antioxidant Capacity in Women With Metabolic Syndrome.” Nutrition Research 31, no. 3: 190–196. 10.1016/j.nutres.2011.02.003. 21481712 PMC3075541

[fsn371562-bib-0005] Bodet, C. , F. Chandad , and D. Grenier . 2006. “Anti‐Inflammatory Activity of a High‐Molecular‐Weight Cranberry Fraction on Macrophages Stimulated by Lipopolysaccharides From Periodontopathogens.” Journal of Dental Research 85, no. 3: 235–239. 10.1177/154405910608500306. 16498070

[fsn371562-bib-0006] Bonifait, L. , and D. Grenier . 2010. “Cranberry Polyphenols: Potential Benefits for Dental Caries and Periodontal Disease.” Journal (Canadian Dental Association) 76: a130. 20943032

[fsn371562-bib-0007] Chew, B. , B. Mathison , L. Kimble , et al. 2019. “Chronic Consumption of a Low Calorie, High Polyphenol Cranberry Beverage Attenuates Inflammation and Improves Glucoregulation and HDL Cholesterol in Healthy Overweight Humans: A Randomized Controlled Trial.” European Journal of Nutrition 58, no. 3: 1223–1235. 10.1007/s00394-018-1643-z. 29476238 PMC6499871

[fsn371562-bib-0008] Choi, J. S. , Y. J. Choi , S. H. Park , J. S. Kang , and Y. H. Kang . 2004. “Flavones Mitigate Tumor Necrosis Factor‐Alpha‐Induced Adhesion Molecule Upregulation in Cultured Human Endothelial Cells: Role of Nuclear Factor‐Kappa B.” Journal of Nutrition 134, no. 5: 1013–1019. 10.1093/jn/134.5.1013. 15113938

[fsn371562-bib-0009] Collett, G. P. , and F. C. Campbell . 2004. “Curcumin Induces c‐Jun N‐Terminal Kinase‐Dependent Apoptosis in HCT116 Human Colon Cancer Cells.” Carcinogenesis 25, no. 11: 2183–2189. 10.1093/carcin/bgh233. 15256484

[fsn371562-bib-0010] Cumpston, M. , T. Li , M. J. Page , et al. 2019. “Updated Guidance for Trusted Systematic Reviews: A New Edition of the Cochrane Handbook for Systematic Reviews of Interventions.” Cochrane Database of Systematic Reviews 10, no. 10: Ed000142. 10.1002/14651858.ed000142. 31643080 PMC10284251

[fsn371562-bib-0011] de Souza Gouveia Moreira, L. , K. T. Resende Teixeira , L. Cardozo , et al. 2024. “Effects of Cranberry Extract (*Vaccinium macrocarpon*) Supplementation on Lipid Peroxidation and Inflammation in Patients With Chronic Kidney Disease (Stages 3‐4): A Randomized Controlled Trial.” Journal of Nutrition and Metabolism 2024: 9590066. 10.1155/2024/9590066. 38752013 PMC11095989

[fsn371562-bib-0012] Denis, M. C. , Y. Desjardins , A. Furtos , et al. 2015. “Prevention of Oxidative Stress, Inflammation and Mitochondrial Dysfunction in the Intestine by Different Cranberry Phenolic Fractions.” Clinical Science (London) 128, no. 3: 197–212. 10.1042/cs20140210. 25069567

[fsn371562-bib-0013] DerSimonian, R. , and N. Laird . 1986. “Meta‐Analysis in Clinical Trials.” Controlled Clinical Trials 7, no. 3: 177–188. 10.1016/0197-2456(86)90046-2. 3802833

[fsn371562-bib-0014] Dohadwala, M. M. , M. Holbrook , N. M. Hamburg , et al. 2011. “Effects of Cranberry Juice Consumption on Vascular Function in Patients With Coronary Artery Disease.” American Journal of Clinical Nutrition 93, no. 5: 934–940. 10.3945/ajcn.110.004242. PMC3076649 21411615

[fsn371562-bib-0015] Duffey, K. J. , and L. A. Sutherland . 2015. “Adult Consumers of Cranberry Juice Cocktail Have Lower C‐Reactive Protein Levels Compared With Nonconsumers.” Nutrition Research 35, no. 2: 118–126. 10.1016/j.nutres.2014.11.005. 25530012

[fsn371562-bib-0016] Eftekhari, M. H. , M. Allaei , S. Khosropanah , A. Rajaeifard , and Z. Sohrabi . 2016. “Cranberry Supplement and Metabolic Risk Factors in Obese and Overweight Females.” Jentashapir Journal of Health Research 7, no. 3: e37255.

[fsn371562-bib-0017] Fatel, E. C. S. , F. T. Rosa , D. F. Alfieri , et al. 2021. “Beneficial Effects of Fish Oil and Cranberry Juice on Disease Activity and Inflammatory Biomarkers in People With Rheumatoid Arthritis.” Nutrition 86: 111183. 10.1016/j.nut.2021.111183. 33636418

[fsn371562-bib-0018] Flammer, A. J. , E. A. Martin , M. Gössl , et al. 2013. “Polyphenol‐Rich Cranberry Juice Has a Neutral Effect on Endothelial Function but Decreases the Fraction of Osteocalcin‐Expressing Endothelial Progenitor Cells.” European Journal of Nutrition 52, no. 1: 289–296. 10.1007/s00394-012-0334-4. 22382203 PMC3943114

[fsn371562-bib-0019] Higgins, J. P. , D. G. Altman , P. C. Gøtzsche , et al. 2011. “The Cochrane Collaboration's Tool for Assessing Risk of Bias in Randomised Trials.” BMJ 343: d5928. 10.1136/bmj.d5928. 22008217 PMC3196245

[fsn371562-bib-0020] Hotamisligil, G. S. 2017. “Inflammation, Metaflammation and Immunometabolic Disorders.” Nature 542, no. 7640: 177–185. 10.1038/nature21363. 28179656

[fsn371562-bib-0021] Hsia, D. S. , D. J. Zhang , R. S. Beyl , F. L. Greenway , and C. Khoo . 2020. “Effect of Daily Consumption of Cranberry Beverage on Insulin Sensitivity and Modification of Cardiovascular Risk Factors in Adults With Obesity: A Pilot, Randomised, Placebo‐Controlled Study.” British Journal of Nutrition 124, no. 6: 577–585. 10.1017/s0007114520001336. 32301407 PMC9014773

[fsn371562-bib-0022] Joseph, S. V. , I. Edirisinghe , and B. M. Burton‐Freeman . 2016. “Fruit Polyphenols: A Review of Anti‐Inflammatory Effects in Humans.” Critical Reviews in Food Science and Nutrition 56, no. 3: 419–444. 10.1080/10408398.2013.767221. 25616409

[fsn371562-bib-0023] Kantor, E. D. , J. W. Lampe , M. Kratz , and E. White . 2013. “Lifestyle Factors and Inflammation: Associations by Body Mass Index.” PLoS One 8, no. 7: e67833. 10.1371/journal.pone.0067833. 23844105 PMC3699492

[fsn371562-bib-0024] Kim, M. J. , J. Y. Chung , J. H. Kim , and H. K. Kwak . 2013. “Effects of Cranberry Powder on Biomarkers of Oxidative Stress and Glucose Control in Db/Db Mice.” Nutrition Research and Practice 7, no. 6: 430–438. 10.4162/nrp.2013.7.6.430. 24353827 PMC3865264

[fsn371562-bib-0025] Kim, M. J. , J. Ohn , J. H. Kim , and H. K. Kwak . 2011. “Effects of Freeze‐Dried Cranberry Powder on Serum Lipids and Inflammatory Markers in Lipopolysaccharide Treated Rats Fed an Atherogenic Diet.” Nutrition Research and Practice 5, no. 5: 404–411. 10.4162/nrp.2011.5.5.404. 22125677 PMC3221825

[fsn371562-bib-0026] Kunnumakkara, A. B. , S. Guha , S. Krishnan , P. Diagaradjane , J. Gelovani , and B. B. Aggarwal . 2007. “Curcumin Potentiates Antitumor Activity of Gemcitabine in an Orthotopic Model of Pancreatic Cancer Through Suppression of Proliferation, Angiogenesis, and Inhibition of Nuclear Factor‐kappaB‐Regulated Gene Products.” Cancer Research 67, no. 8: 3853–3861. 10.1158/0008-5472.Can-06-4257. 17440100

[fsn371562-bib-0027] Latruffe, N. 2017. “Natural Products and Inflammation.” Molecules 22, no. 1: 120. 10.3390/molecules22010120. 28085099 PMC6155884

[fsn371562-bib-0028] Lee, I. T. , Y. C. Chan , C. W. Lin , W. J. Lee , and W. H. Sheu . 2008. “Effect of Cranberry Extracts on Lipid Profiles in Subjects With Type 2 Diabetes.” Diabetic Medicine 25, no. 12: 1473–1477. 10.1111/j.1464-5491.2008.02588.x. 19046248

[fsn371562-bib-0029] Leitão, D. P. , A. C. Polizello , I. Y. Ito , and A. C. Spadaro . 2005. “Antibacterial Screening of Anthocyanic and Proanthocyanic Fractions From Cranberry Juice.” Journal of Medicinal Food 8, no. 1: 36–40. 10.1089/jmf.2005.8.36. 15857207

[fsn371562-bib-0030] Libby, P. 2006. “Inflammation and Cardiovascular Disease Mechanisms.” American Journal of Clinical Nutrition 83, no. 2: 456s–460s. 10.1093/ajcn/83.2.456S. 16470012

[fsn371562-bib-0031] Liberati, A. , D. G. Altman , J. Tetzlaff , et al. 2009. “The PRISMA Statement for Reporting Systematic Reviews and Meta‐Analyses of Studies That Evaluate Health Care Interventions: Explanation and Elaboration.” Journal of Clinical Epidemiology 62, no. 10: e1–e34. 10.1016/j.jclinepi.2009.06.006. 19631507

[fsn371562-bib-0032] Mathison, B. D. , L. L. Kimble , K. L. Kaspar , C. Khoo , and B. P. Chew . 2014. “Consumption of Cranberry Beverage Improved Endogenous Antioxidant Status and Protected Against Bacteria Adhesion in Healthy Humans: A Randomized Controlled Trial.” Nutrition Research 34, no. 5: 420–427. 10.1016/j.nutres.2014.03.006. 24916555

[fsn371562-bib-0033] McKay, D. L. , and J. B. Blumberg . 2007. “Cranberries (*Vaccinium macrocarpon*) and Cardiovascular Disease Risk Factors.” Nutrition Reviews 65, no. 11: 490–502. 10.1111/j.1753-4887.2007.tb00273.x. 18038941

[fsn371562-bib-0034] McKay, D. L. , C. Y. Chen , C. A. Zampariello , and J. B. Blumberg . 2015. “Flavonoids and Phenolic Acids From Cranberry Juice Are Bioavailable and Bioactive in Healthy Older Adults.” Food Chemistry 168: 233–240. 10.1016/j.foodchem.2014.07.062. 25172705

[fsn371562-bib-0035] Medzhitov, R. 2010. “Inflammation 2010: New Adventures of an Old Flame.” Cell 140, no. 6: 771–776. 10.1016/j.cell.2010.03.006. 20303867

[fsn371562-bib-0036] Moss, S. F. , and M. J. Blaser . 2005. “Mechanisms of Disease: Inflammation and the Origins of Cancer.” Nature Clinical Practice Oncology 2, no. 2: 90–97. 10.1038/ncponc0081. 16264881

[fsn371562-bib-0037] Neto, C. C. 2007. “Cranberry and Blueberry: Evidence for Protective Effects Against Cancer and Vascular Diseases.” Molecular Nutrition & Food Research 51, no. 6: 652–664. 10.1002/mnfr.200600279. 17533651

[fsn371562-bib-0038] Novotny, J. A. , D. J. Baer , C. Khoo , S. K. Gebauer , and C. S. Charron . 2015. “Cranberry Juice Consumption Lowers Markers of Cardiometabolic Risk, Including Blood Pressure and Circulating C‐Reactive Protein, Triglyceride, and Glucose Concentrations in Adults.” Journal of Nutrition 145, no. 6: 1185–1193. 10.3945/jn.114.203190. 25904733

[fsn371562-bib-0039] Pahl, H. L. 1999. “Activators and Target Genes of Rel/NF‐kappaB Transcription Factors.” Oncogene 18, no. 49: 6853–6866. 10.1038/sj.onc.1203239. 10602461

[fsn371562-bib-0040] Paul, S. , H. S. Shin , and S. C. Kang . 2012. “Inhibition of Inflammations and Macrophage Activation by Ginsenoside‐Re Isolated From Korean Ginseng ( *Panax ginseng* C.A. Meyer).” Food and Chemical Toxicology: An International Journal Published for the British Industrial Biological Research Association 50, no. 5: 1354–1361. 10.1016/j.fct.2012.02.035. 22401937

[fsn371562-bib-0041] Richter, C. K. , A. C. Skulas‐Ray , T. L. Gaugler , S. Meily , K. S. Petersen , and P. M. Kris‐Etherton . 2021. “Effects of Cranberry Juice Supplementation on Cardiovascular Disease Risk Factors in Adults With Elevated Blood Pressure: A Randomized Controlled Trial.” Nutrients 13, no. 8: 2618. 10.3390/nu13082618. 34444779 PMC8398037

[fsn371562-bib-0042] Roy, S. , S. Khanna , H. M. Alessio , et al. 2002. “Anti‐Angiogenic Property of Edible Berries.” Free Radical Research 36, no. 9: 1023–1031. 10.1080/1071576021000006662. 12448828

[fsn371562-bib-0043] Ruel, G. , S. Pomerleau , P. Couture , S. Lemieux , B. Lamarche , and C. Couillard . 2008. “Low‐Calorie Cranberry Juice Supplementation Reduces Plasma Oxidized LDL and Cell Adhesion Molecule Concentrations in Men.” British Journal of Nutrition 99, no. 2: 352–359. 10.1017/s0007114507811986. 17761017

[fsn371562-bib-0044] Santangelo, C. , R. Varì , B. Scazzocchio , R. Di Benedetto , C. Filesi , and R. Masella . 2007. “Polyphenols, Intracellular Signalling and Inflammation.” Annali Dell'istituto Superiore di Sanita 43, no. 4: 394–405. 18209273

[fsn371562-bib-0045] Shahavandi, M. , M. R. Amini , H. Shahinfar , and S. Shab‐Bidar . 2021. “Major Dietary Patterns and Predicted Cardiovascular Disease Risk in an Iranian Adult Population.” Nutrition and Health 27, no. 1: 27–37. 10.1177/0260106020952591. 32867574

[fsn371562-bib-0046] Shoelson, S. E. , J. Lee , and A. B. Goldfine . 2006. “Inflammation and Insulin Resistance.” Journal of Clinical Investigation 116, no. 7: 1793–1801. 10.1172/jci29069. PMC1483173 16823477

[fsn371562-bib-0047] Simão, T. N. , M. A. Lozovoy , A. N. Simão , et al. 2013. “Reduced‐Energy Cranberry Juice Increases Folic Acid and Adiponectin and Reduces Homocysteine and Oxidative Stress in Patients With the Metabolic Syndrome.” British Journal of Nutrition 110, no. 10: 1885–1894. 10.1017/s0007114513001207. 23750500

[fsn371562-bib-0048] Sterne, J. A. C. , J. Savović , M. J. Page , et al. 2019. “RoB 2: A Revised Tool for Assessing Risk of Bias in Randomised Trials.” BMJ (Clinical Research Ed.) 366: l4898. 10.1136/bmj.l4898. 31462531

[fsn371562-bib-0049] Sun, J. , and R. Hai Liu . 2006. “Cranberry Phytochemical Extracts Induce Cell Cycle Arrest and Apoptosis in Human MCF‐7 Breast Cancer Cells.” Cancer Letters 241, no. 1: 124–134. 10.1016/j.canlet.2005.10.027. 16377076

[fsn371562-bib-0050] Tsai, D. H. , M. Riediker , A. Berchet , et al. 2019. “Effects of Short‐ and Long‐Term Exposures to Particulate Matter on Inflammatory Marker Levels in the General Population.” Environmental Science and Pollution Research International 26, no. 19: 19697–19704. 10.1007/s11356-019-05194-y. 31079306

[fsn371562-bib-0051] Vinson, J. A. , P. Bose , J. Proch , H. Al Kharrat , and N. Samman . 2008. “Cranberries and Cranberry Products: Powerful In Vitro, Ex Vivo, and In Vivo Sources of Antioxidants.” Journal of Agricultural and Food Chemistry 56, no. 14: 5884–5891. 10.1021/jf073309b. 18558697

[fsn371562-bib-0052] Wang, M. , J. Liu , Z. Zhang , et al. 2022. “Effects of Dietary Intervention on Inflammatory Markers in Metabolic Syndrome: A Systematic Review and Meta‐Analysis.” Frontiers in Nutrition 9: 846591. 10.3389/fnut.2022.846591. PMC9008568 35433780

[fsn371562-bib-0053] Weiss, E. I. , Y. Houri‐Haddad , E. Greenbaum , N. Hochman , I. Ofek , and Z. Zakay‐Rones . 2005. “Cranberry Juice Constituents Affect Influenza Virus Adhesion and Infectivity.” Antiviral Research 66, no. 1: 9–12. 10.1016/j.antiviral.2004.12.011. 15781126

[fsn371562-bib-0054] Wellen, K. E. , and G. S. Hotamisligil . 2005. “Inflammation, Stress, and Diabetes.” Journal of Clinical Investigation 115, no. 5: 1111–1119. 10.1172/jci25102. 15864338 PMC1087185

[fsn371562-bib-0055] Zhu, Y. , M. Xia , Y. Yang , et al. 2011. “Purified Anthocyanin Supplementation Improves Endothelial Function via NO‐cGMP Activation in Hypercholesterolemic Individuals.” Clinical Chemistry 57, no. 11: 1524–1533. 10.1373/clinchem.2011.167361. 21926181

